# Influence of Alumina Addition on the Optical Properties and the Thermal Stability of Titania Thin Films and Inverse Opals Produced by Atomic Layer Deposition

**DOI:** 10.3390/nano11041053

**Published:** 2021-04-20

**Authors:** Martin Waleczek, Jolien Dendooven, Pavel Dyachenko, Alexander Y. Petrov, Manfred Eich, Robert H. Blick, Christophe Detavernier, Kornelius Nielsch, Kaline P. Furlan, Robert Zierold

**Affiliations:** 1 Institute of Nanostructure and Solid State Physics & Center for Hybrid Nanostructures, Universität Hamburg, Luruper Chausse 149, 22761 Hamburg, Germany; mwalecze@bynt.de (M.W.); rblick@physnet.uni-hamburg.de (R.H.B.); 2 COCOON Group, Department of Solid State Sciences, Ghent University, Krijgslaan 281/S1, B-9000 Ghent, Belgium; Jolien.Dendooven@UGent.be (J.D.); Christophe.Detavernier@UGent.be (C.D.); 3 Holoeye Photonics AG, Volmerstrasse 1, 12489 Berlin, Germany; p.n.dyachenko@gmail.com; 4 Institute of Optical and Electronic Materials, Hamburg University of Technology, Eißendorfer Str. 38, 21073 Hamburg, Germany; a.petrov@tuhh.de (A.Y.P.); m.eich@tuhh.de (M.E.); 5 Institute of Hydrogen Technology, Helmholtz-Zentrum hereon, Max-Planck-Straße 1, D-21502 Geesthacht, Germany; 6 Institute of Materials Science, Technical University Dresden, Helmholtzstr. 10, 01069 Dresden, Germany; k.nielsch@ifw-dresden.de; 7 IFW Dresden, Institute for Metallic Materials, Helmholtzstr. 20, 01069 Dresden, Germany; 8 Institute of Advanced Ceramics, Hamburg University of Technology, Denickest. 15, 21073 Hamburg, Germany

**Keywords:** atomic layer deposition, optical properties, inverse opal photonic crystals, bio-inspired materials, ceramics, high-temperature stability

## Abstract

TiO_2_ thin films deposited by atomic layer deposition (ALD) at low temperatures (<100 °C) are, in general, amorphous and exhibit a smaller refractive index in comparison to their crystalline counterparts. Nonetheless, low-temperature ALD is needed when the substrates or templates are based on polymeric materials, as the deposition has to be performed below their glass transition or melting temperatures. This is the case for photonic crystals generated via ALD infiltration of self-assembled polystyrene templates. When heated up, crystal phase transformations take place in the thin films or photonic structures, and the accompanying volume reduction as well as the burn-out of residual impurities can lead to mechanical instability. The introduction of cation doping (e.g., Al or Nb) in bulk TiO_2_ parts is known to alter phase transitions and to stabilize crystalline phases. In this work, we have developed low-temperature ALD super-cycles to introduce Al_2_O_3_ into TiO_2_ thin films and photonic crystals. The aluminum oxide content was adjusted by varying the TiO_2_:Al_2_O_3_ internal loop ratio within the ALD super-cycle. Both thin films and inverse opal photonic crystal structures were subjected to thermal treatments ranging from 200 to 1200 °C and were characterized by in- and ex-situ X-ray diffraction, spectroscopic ellipsometry, and spectroscopic reflectance measurements. The results show that the introduction of alumina affects the crystallization and phase transition temperatures of titania as well as the optical properties of the inverse opal photonic crystals (iPhC). The thermal stability of the titania iPhCs was increased by the alumina introduction, maintaining their photonic bandgap even after heat treatment at 900 °C and outperforming the pure titania, with the best results being achieved with the super-cycles corresponding to an estimated alumina content of 26 wt.%.

## 1. Introduction

Photonic crystals and glasses are periodic or disordered arrangements of refractive index perturbations. They are present in natural materials such as bird feathers, butterfly wings, and beetle wing-cases, presenting a so-called structural color. The color arises from the interference of the electromagnetic radiation at their structure, which causes the reflection of certain colors rather than absorption or pigmentation [1]. Bio-inspired synthetic photonic crystals and glasses make use of this selectivity in the reflection of the spectrum, while expanding the spectral range to ultraviolet and infrared. Thus, applications not only as structural colors [2,3,4,5,6] but also as broadband reflectors [7], optical filters, thermal emitters for thermophotovoltaics [8], and in radiative cooling [9,10] are demonstrated. In a broader context, photonic crystals and glasses based on inversion of colloidal templates (also referred to as colloidal-based porous materials) can be used as sensors, membranes, self-cleaning surfaces, and in batteries and water purification systems [11]. Such “inverted” structures are identified by many different names, but the fabrication processes for all of them basically involve the coating or infiltration of a sacrificial template with a polymeric, metallic, or ceramic material [1,11,12]. The infiltration step of the sacrificial template can be performed by sol–gel, colloidal routes, or thin-film deposition techniques, such as atomic layer deposition (ALD). Upon template removal, e.g., by burn-out for polymers or HF-etching for silica, an inverted structure is created in which macropores are either periodically arranged or disordered, depending on whether the initial template had a crystal or a glass-like structure, respectively. When these synthetic structures are periodically ordered, they present a photonic bandgap, i.e., a narrow spectral region in which the propagation of electromagnetic radiation is ‘forbidden’, or, in other words, reflection is enhanced. Since the photonic bandgap depends on the interaction of the radiation with the geometrical structure, it can be tuned by alterations in the synthetic photonic crystal structure, such as the initial template size (macropore size after inversion), refractive index of the (infiltrating) material, and thickness of the shell. In this sense, ALD provides a unique capability of both material and thickness alteration, whilst being capable of conformally infiltrating high-aspect-ratio structures.

Atomic layer deposition is a process based on self-limiting surface-controlled chemical reactions between vaporized precursors and substrate, capable of producing a wide variety of materials comprising sulfides, selenides, tellurides, metals, nitrides, and oxides [13]. ALD has already been demonstrated for the production of inverse opal photonic crystals (iPhC) made from gallium phosphide [14], zinc sulfide [15], tungsten [8], zinc oxide [16,17], aluminum oxide [17,18,19], aluminosilicates [20,21], and titanium oxide [17,22,23,24]. In particular, the latter one is of interest for optical applications since titania features a relatively high refractive index, often higher than 2.3 at 632.8 nm. The exact refractive index value is known to vary depending on the deposition temperature and the ALD precursor used to grow the film [25,26,27,28]. Moreover, it also depends on the crystalline phase, as well as the overall film density [29]. In this sense, the rutile phase is desirable, as it presents higher refractive index values up to 2.6, reported for sputtered films [30]. 

Rutile is the most stable polymorph of titanium oxide phases and often found at high temperatures or after high-temperature exposure, as the metastable phases anatase and brookite transform into rutile. The anatase to rutile phase transformation is accompanied by an expansion of the tetragonal structure in a direction and a shrinkage in the c direction. Overall, the unit cell volume is substantially reduced (0.1363 to 0.0624 nm^3^) and the density increased (3.8 to 4.2 g/cm^3^) [31]. This transition can lead, however, to instability and cracks in films and nanostructures [22]. Furthermore, whilst the rutile phase is desired due to its high refractive index, the anatase phase is often desired for its higher activity in photocatalytic applications [32]. As a consequence, there is a strong need to exactly control the phase transformation of titanium oxide to either assure the presence of a phase, needed for a specific target application, or to avoid undesirable phase transformation upon service. 

In powder metallurgy processing, dopants are often used to influence the sintering behavior of powder compacts as well as to reduce grain boundary diffusivity and to improve the mechanical properties. These tailored properties can be achieved already at very low doping amounts, such as 0.1 mol.%, and have been widely demonstrated for aluminum oxide matrixes [33,34]. The tailoring of phase transformations can also be achieved by introducing certain cations within the host lattice. Such an approach has been demonstrated for titanium oxide gels that were doped with a variety of cations (Na, Co, Zn, La, Al, Ca, K, and Ba), all of which influenced the anatase-to-rutile phase transition temperature, depending on their ionic radius and charge [35]. A shift in the phase transformation temperature was also reported when doping ALD films [36] and laser-induced synthesized titanium oxide powders [37,38] with niobium. Nonetheless, the controlled addition of dopants is often challenging, especially at nanostructures. In this regard, the sol–gel route is limited in the capability of insertion of high-doping amount, as segregation is likely to occur [39]. Moreover, sol–gel films are often prone to cracking upon drying and, in the case of multicomponent films based on alkoxide precursors, different reactivity could lead to unwanted precipitation of monophasic phases rather than an homogeneous mix [40,41,42]. Meanwhile, ALD is capable of fabricating a wide composition range of mixed structures, as the doping occurs cycle by cycle, layer by layer, with thickness control down to the sub-nm range. In detail, the fabrication of dual structures or ternary oxides is realized via so-called super cycling of two binary cycles and was already demonstrated for titanium oxide and aluminum oxide cycles as planar films onto Ru-coated Si wafers [43]. Furthermore, ALD offers the possibility of infiltrating high-aspect-ratio structures, such as the iPhC polymeric templates, even at low temperatures, either with single oxides or ternary oxides in a super-cycle approach [20]. 

In this work, we have investigated the influence of the addition of alumina on the optical properties and the phase transformation of titanium oxide films. We demonstrate the infiltration of iPhC templates with alumina–titanium oxide films at a very low deposition temperature (95 °C). A wide range of aluminum oxide content (from 4 up to 63 wt.%) as well as full-mix structures (1:1 super-cycling) was realized. As such, the superior optical properties of TiO_2_ and superior mechanical properties of Al_2_O_3_ were combined, resulting in an iPhC with high refractive index (2.16 for 5 wt.% of aluminum oxide), being capable of maintaining its 3D periodic structure even after high-temperature exposure. In situ XRD measurements show that, for alumina contents between 16 and 32 wt.%, the formation of anatase is suppressed and the films crystallize directly into the rutile phase. The optimum aluminum oxide amount regarding both phase stabilization and optical properties lies within the range of 8–26 wt.%, at which the alumina-doped iPhC presents a well-defined photonic bandgap even after exposure at 900 °C.

## 2. Materials and Methods 

**Figure 1:**
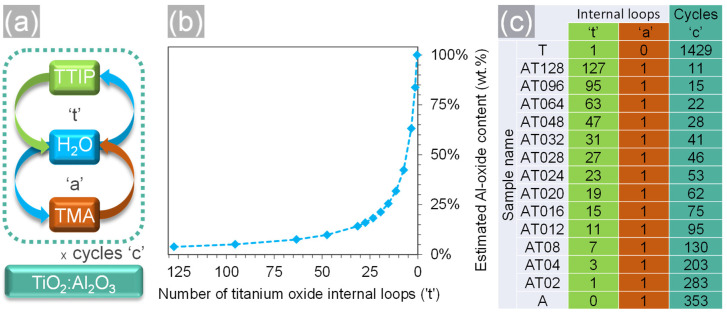
ALD super-cycle processes used to deposit titanium oxide doped with aluminum oxide films. (**a**) Schematic illustration of ALD super-cycles showing the internal loops for titania (‘t’) and alumina (‘a’) as well as the outer loop for the cycling (‘c’). All three define the content of oxides within the film, depicted in (**b**). An overview of all samples produced within this study and their respective super-cycle parameters is shown in (**c**).

ALD thin films were deposited in a home-made ALD reactor (Universität Hamburg, Physics Department, CHyN) using a super-cycle approach (Figure 1a), in which the ratio TiO_2_:Al_2_O_3_ was tailored by varying the internal loop number (‘t’ and ‘a’) and the total cycle number (‘c’), while keeping the target thickness constant (~50 nm). The oxide content ratio within the film (Figure 1b) was estimated based on the reported film densities [44,45,46] and the growth per cycle (GPC) of the individual oxides determined on planar references, which were 0.35 and 1.42 Angstroms for titania and alumina, respectively. The precursors used to deposit Al_2_O_3_ and TiO_2_ layers were trimethylaluminum (min. 98% TMA, Strem chemicals) and titanium isopropoxide (>98% TTIP, Sigma-Aldrich), which were cycled with deionized water under a constant flow of 30 sccm nitrogen (carrier gas). The system’s base pressure without nitrogen flow was 9 × 10^−2^ mbar and, during the process, it was 5 × 10^−1^ mbar. TTIP was heated up to 80 °C while water and TMA were kept at room temperature. For planar films, <100> silicon wafers with a 300 nm SiO_2_ layer were used as substrates, and, for the photonic crystals’ fabrication, a polymeric template was used, which is described later. For both types of substrates, full exposure mode was used and the parameters for pulse, exposure, and pump time were 0.2/60/90 s and 1/60/90 s for TMA, H_2_O, and TTIP, respectively.

The thickness and refractive index of the resulting films were determined before and after heat treatments by spectroscopic ellipsometry (SENProTM, SENTECH Instruments GmbH, Jena, Germany) at an incident angle of 70° and using a custom Cauchy model for the fitting procedure. X-ray diffraction (XRD) was used to access the crystallization behavior of the deposited films according to the heat treatment temperatures and aluminum oxide content. The XRD measurements were performed on a Bruker D8 diffractometer with Cu Kα radiation and equipped with a custom-built heating chamber. Two types of measurements were performed: on the one hand, in situ XRD measurements in the 2θ range of 21–37°, relevant for titanium oxide phases, were conducted while heating the samples in air from room temperature up to 900 or 1000 °C with a constant heating rate of 15 °C/min. A linear detector covering 20° in 2θ was used to collect the diffraction data with a 2 s time resolution. On the other hand, a full scan in 2θ (20–60°) was performed after each in situ annealing experiment. 

Vertical convective self-assembly was used to fabricate direct opaline polymeric templates of monodisperse polystyrene particles with particle diameter of 522 ± 15 nm (Microparticles GmbH, Berlin Germany) onto sapphire substrates (Crystec GmbH, Berlin Germany). The received suspensions were diluted with deionized filtered water (di-H_2_O) down to 1 mg/mL inside polytetrafluorethylene (PTFE) beakers. PTFE was used to avoid the assembly of particles onto the beaker walls. Prior to immersion into the suspensions, the substrates were cleaned for 60 min in an ultrasonic bath containing an alkaline detergent (Mucasol 1% solution, Merz Hygiene GmbH, Norderstedt, Germany), brushed, rinsed with di-H_2_O, dried with nitrogen gas, and further activated via a 20 min oxygen plasma treatment (Polaron PT7160, Quorum Technologies, Sussex, UK). The self-assembly took place inside a humidity chamber (HCP 108, Memmert GmbH, Schwabach, Germany) at 55 °C and relative humidity of 70–80% for several days until the substrates were covered with polymeric opaline films. Such colloidal films, also called direct photonic crystals, were coated (and the 3D structure was then “infiltrated”) via ALD using the same super-cycle approach depicted in Figure 1 and the corresponding paragraph above. The infiltration temperature was kept at 95 °C to avoid damage of the polystyrene template, which had a glass transition temperature around 105 °C [47].

After ALD, the polymeric template was burned-out at 500 °C in a tubular furnace at ambient atmosphere (air) for 30 min, generating the inverse opal photonic crystals. Further heat treatments on both films deposited on silicon wafer and inverse opal photonic crystals were also performed in a tubular furnace and the maximum temperature was varied between 200 and 1200 °C. The dwell time and the heating rate for all cycles were 60 min and 1 °C/min, respectively. The morphology of the inverse opal photonic crystals was analyzed via scanning electron microscopy (SEM, Zeiss Sigma, Oberkochen, Germany) and their reflectance was measured with incident angle of 8° in a UV–vis–NIR spectrometer (Lambda 1050, Perkin-Elmer, Waltham, Massachusetts, USA) and collected in the 0.7–1.6 µm wavelength range for a 5 × 5 mm^2^ area, before and after heat treatments. After acquisition, the data were analyzed and smoothed with a 6-point adjacent averaging on Origin Lab 2020©.

## 3. Results and Discussion

### 3.1. Refractive Index Evolution

**Figure 2:**
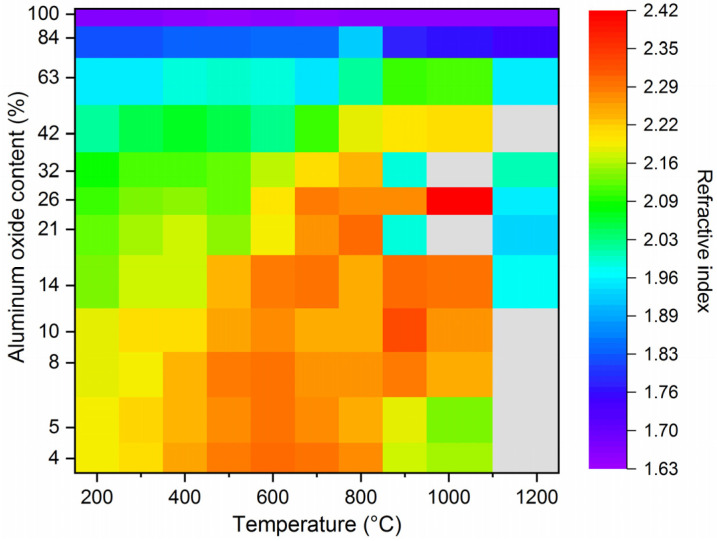
Evolution of refractive indexes of titania thin films according to the alumina content and annealing temperature ranging from 200 to 1200 °C with a dwell time of 60 min and a heating rate of 1 °C/min. Grey boxes indicate measurements at which the fit in spectroscopic analyses did not converge, indicating discontinuous thin films.

**Figure 3:**
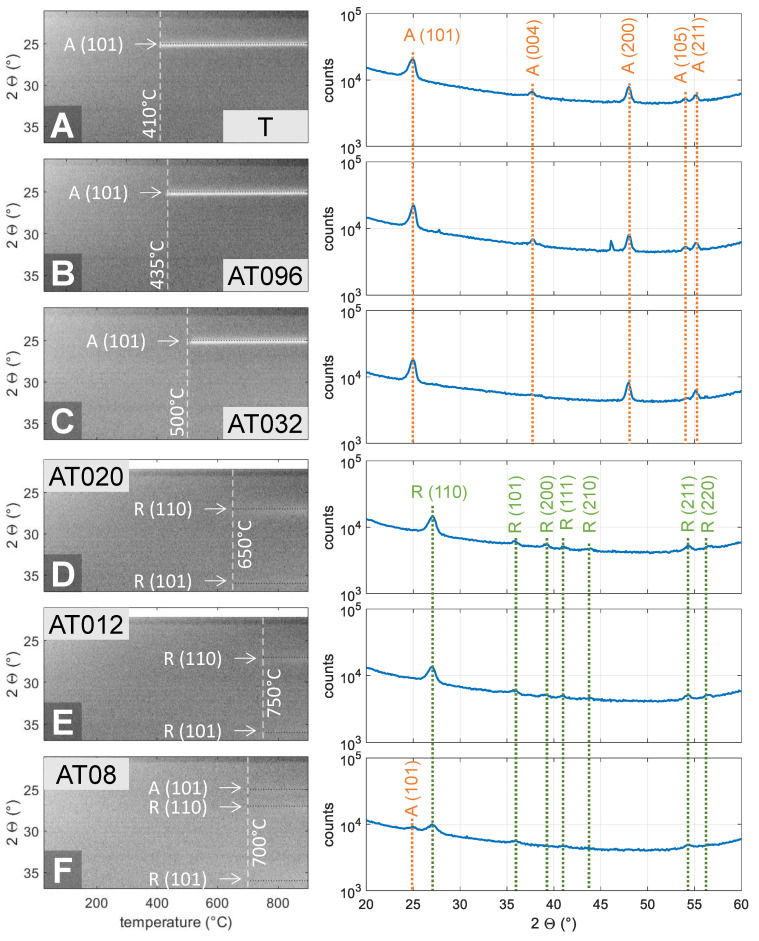
XRD in situ measurements from RT up to 900 °C (0.25°C/s) and full-range measurements after reaching the final temperature. Phases and the respective planes for each peak are shown, where ‘A’ stands for anatase (PDF 01-070-8501) and ‘R’ for rutile (PDF 01-072-7374). Estimated aluminum oxide content is (**a**) 0%, (**b**) 5%, (**c**) 14%, (**d**) 21%, (**e**) 32%, and (**f**) 42%, respectively. To enhance visualization, only selected compositions are shown. Please note that the axes have slightly different ranges for (a,b,c,f) and (d,e). For a full version, please refer to Figure S3.

**Figure 4:**
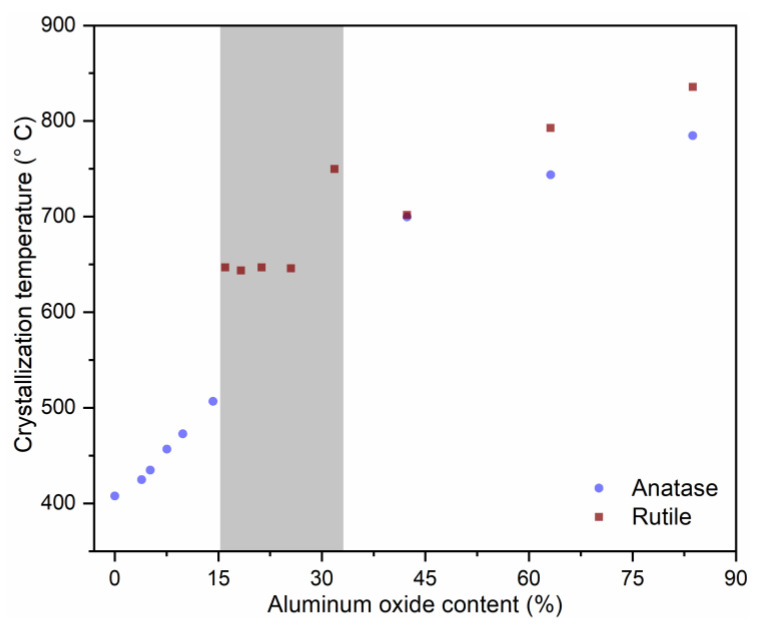
Influence of aluminum oxide content on the crystallization behavior and temperature of titanium dioxide phases, namely anatase and rutile. Data points were extracted from the in situ XRD measurements performed up to 900 °C, shown in Figure 3 and Figure S3. The grey area indicates the region for which the films crystallize directly into rutile phase. Note that after heat treatment at 1200 °C, all films present the rutile phase; see Figure S2 for details.

**Figure 5:**
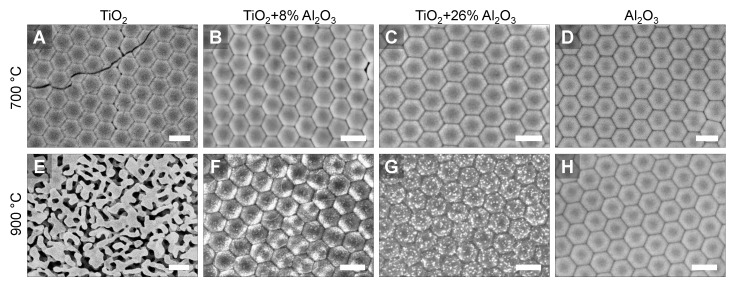
Comparison between the top view morphology of inverse opal photonic crystals with different TiO_2_ to Al_2_O_3_ ratio imaged by SEM (inLens detector) after heat treatments performed at (**a–d**) 700 °C and (**e–h**) 900 °C for 1 h. Estimated Al_2_O_3_ content in weight percentage is (a,e) 0 %, (b,f) 8%, (c,g) 26%, and (d,h) 100%. Scale bars correspond to 500 nm.

The refractive indexes after ALD for the full titania and full alumina sample amounted to n = 2.187 ± 0.012 and 1.635 ± 0.002 (@632.8 nm), respectively, being in agreement with earlier reports [18,19,20,21,22,23,47,48,49,50,51,52]. All the other super-cycle samples fell within this interval (Figure 2), indicating that the concept of tailor-made mixing of the oxides by variation of the sub-cycle ratio can be applied successfully. Upon heat treatment, all ternary compositions as well as the pure TiO_2_ thin film showed an increase in the refractive index, which can be related to two factors: on the one hand, heat treatments can promote the removal of common film impurities, such as nitrogen, carbon, as well as hydrogen and could lead to further densification of the thin film. Such a process could explain the slight increase in the refractive index observed at lower temperatures [53,54]. On the other hand, the annealing of ALD films led to crystallization of phases within the film, discussed in detail in the XRD section, which have higher refractive indexes than the as-deposited amorphous thin films [26]. Noticeably, the temperature at which the highest refractive index is reached significantly shifts to higher temperatures with increasing aluminum oxide content (compare, for example, the sample with 4 wt.% and 20 wt.% of alumina). We attribute this peak shift to an undergone phase transformation from amorphous to anatase or rutile, respectively (discussed in detail in the ‘3.2. Phase Transition and Thermal Stability’ section). Nonetheless, most of the samples showed a measurable decrease in the refractive index after annealing at temperatures above 900 °C, which indicates sample degradation related to the films’ destabilization, cracking, and sintering. The highest refractive index value is achieved by the sample containing 26 wt.% of alumina, showing a refractive index value of 2.412 after heat treatment at 1000 °C, associated with the direct crystallization into the rutile phase at around 650 °C (as revealed by in situ XRD measurements in Figure 3 and Figure 4) and enhanced thermal stability (Figure 5). After heat treatment at 1200 °C, all samples show a significant change in morphology (Figure S1)—discontinued thin film—which is reflected in a reduction in the apparent refractive index. 

### 3.2. Phase Transition and Thermal Stability

The in situ XRD analysis (Figure 3) confirmed that both the crystallization temperature—from amorphous to the crystalline phase—and phase transition temperature—from anatase to rutile—are influenced by the alumina addition and generally increase with the aluminum oxide content (Figure 4). Due to the super-cycling scheme performed in this work, where one cycle of alumina was performed within several titania cycles (see Figure 1c), such films could be considered, at least locally, as doped films or solid solutions. There will be of course a limit for which the alumina content is quite high in relation to the titania content and, thereby, phase separation will occur, as discussed later. Note all films are kept amorphous up to high temperatures, e.g., 500 °C for sample AT032 (14% of alumina), similar to earlier reports of sol–gel-based compositions [55]. The crystallization is observed by the appearance of a peak in the temperature-dependent in situ XRD measurements. Furthermore, the crystallization temperature matches the temperature at which the refractive index of the thin films starts to rise. However, amorphous films present a lower refractive index than their crystalline counterparts [25,26,27,30] and are therefore less sought for application as photonic crystal-based reflectors, but they are interesting for applications such as antibacterial films or oxygen sensors [56]. Moreover, the compositions with less than 14% of alumina, including the pure titania one, could be attractive for photocatalysis applications, as the anatase phase often exhibits higher photocatalytic activity than the rutile phase [54,55]. 

Ex situ measurements performed after heat treatment at 1200 °C have shown, however, that these and other films transform indeed into rutile (Figure S2), the most stable phase thermodynamically [31,57] being in agreement with earlier studies [32,35]. This temperature is, nonetheless, a very high temperature for phase transformation when compared to other studies, which often show transformation temperatures in the range of 465–775 °C [31,35,57]. The work of Ottermann et al. showed that the phase transformation temperature is directly related to the density of TiO_2_ [58]. ALD is well-known to produce dense, pin-hole-free films compared to other preparation methods, such as sol–gel-based approaches. Thus, the observed increased transformation temperature when in comparison to other studies might be explained by the thin film properties. Furthermore, one reason that rutile is not observed in the in situ measurements might be related to the relatively high heating rate of 15 °C/min and no holding time, as this transformation is known to be affected by this and other factors, such as impurities and sample morphology (bulk vs. film) [31]. In fact, high transformation temperatures have also been observed for nanoparticles generated via metalorganic chemical vapor deposition [59], as well as iPhC nanostructures produced via sol–gel [60]. Moreover, Pasquarelli et al. showed that titania inverse opals annealed in air at 1300 °C for 1 h remain in the anatase phase, whereas elongating the annealing duration to 3 h leads to a complete phase transformation into rutile [22]. This time dependence underlines the involved kinetics during the phase transformation. Thereby, we attribute the high transformation temperatures of this work both to the nanostructure of the thin films and, for the samples with aluminum oxide addition, also the doping of aluminum cations in the titanium oxide crystal structure.

The reason that only rutile is formed for compositions between 16 and 32 wt.% of alumina may be associated with the nucleation and growth of both phases as well as its kinetics. Thermodynamics indicates the rutile phase as more stable. Hence, its formation over anatase should not be considered a surprise, but rather the expected behavior. Nonetheless, several different reports indicate the formation of anatase over rutile when the structures are nanosized, either for sol–gel-based structures [61,62] or powders [63]. We hypothesize that our observation of an inhibition of anatase formation and a direct observation of the rutile phase for compositions with 16–32 wt.% of alumina may be related to several factors, which are concurrent with one another: upon heating, the films receive thermal energy, which can be transferred basically in diffusion, crystal nucleation, crystal growth, and phase transformation. Several authors have reported that the presence of doping atoms or annealing under reducing atmospheres (H_2_) promotes the generation of oxygen vacancies [64,65]. Furthermore, annealing in inert atmospheres such as argon accelerates the transformation rate in comparison to annealing in air, as the oxygen vacancies cannot be replenished [65]. In this work, the diffusion of Al^3+^ cations into the TiO_2_ lattice will promote the generation of oxygen vacancies, while the heat treatment in air promotes vacancy elimination. Since the transformation is a reconstructive one, i.e., it involves the breakage of bonds and rearrangement of the crystal lattice, the transformation is facilitated in a lattice where the oxygen framework is distorted and bonds are already broken. 

Nevertheless, a direct comparison between this work and earlier reports cannot be made, as the majority of the works here cited have studied the transformation of anatase to rutile under several different conditions [57,59,61,62,63,65,66,67] or have reported the crystallization of anatase from amorphous films [35,55,60,68,69,70]. In the case of the films in the range of 16-32 wt.%, no phase transformation is present, but rather a direct crystallization into the rutile phase. A similar behavior was observed by Rafieian et al. [71] on magnetron-sputtered films heat-treated in air. The authors have reported the crystallization of the as-deposited amorphous films directly into the rutile phase for substoichiometric films (TiO_x<2_) in comparison to stoichiometric ones. The authors associated the direct transformation into rutile to the excess oxygen vacancies existent in the substoichiometric films. ALD-based oxide films should, by definition, be stoichiometric, which is related to the nature of the ALD process, based on complete reactions between precursors and surface groups. We have shown in a previous work [72], however, that the growth of ALD titania onto alumina, and vice versa, is affected depending on the underlying layer (alumina or titania). Similar results were obtained by Kim et al. [43]. Thereby, a relation between the results obtained by Rafieian et al. and our results can be found, considering that the oxygen vacancies here are generated by the aluminum oxide addition. This behavior is existent in all films and, as a general rule, the number of generated vacancies increases with the aluminum oxide content [35,68], peaking in the range of 16–32 wt.%.

Above this range, the films crystallize into a mix of rutile and anatase phases, which is not desired for the photonic application considered in this work, as the photonic crystals would present a local variation in refractive index. Moreover, such samples also present peaks in the background which could be associated with alumina transition phases, namely δ-aluminum oxide (PDF patterns 00-046-1131 and 00-046-1215). As a consequence, such samples represent not an alumina-doped titania structure, but rather a multi-phase structure. Note that we expected such a phase separation for higher alumina loadings but wanted to cover and to explore the crystallization behavior in the whole range, i.e., from full titania up to full alumina, to highlight the capability of super-cycle ALD processing. The addition of titania as a second phase in alumina has been demonstrated to improve the mechanical properties of powder metallurgy products [73] and also increase thermal insulation [74]. Of course, these properties are not the focus of this work, but photonic crystal structures have also been reported to be excellent thermal insulators [75] and mechanical meta materials [76]. Due to the high alumina content, we expect that the phase separation occurs even prior to crystallization, with the possible formation of separated clusters of alumina and titania within the film, with the alumina clusters acting as a physical barrier to phase nucleation and growth, as well as phase transformation. Where alumina clusters exist, the titania phase cannot nucleate from amorphous to anatase and, upon anatase nucleation, cannot properly grow if surrounded by alumina clusters. Such cluster formation also affects the phase transformation from anatase to rutile. In this case, alumina clusters will be bonded to the titania clusters and, at these interfaces, the reconstructive phase transformation, which includes the breakage and rearrangement of chemical bonds, is hindered. Lastly, the phase transformation is associated with an ≈ 8% shrinkage and the whole structure needs to accommodate this shrinkage, including the stiffer alumina clusters. An earlier study by Kumar et al. [61] has reported a 300 °C shift to higher temperatures for the phase transformation of titania sol–gel-based membranes, due to the constraint and, thus, stress imposed by α-alumina supports. The authors associate the delayed transformation with the slower rate of nucleation caused by the stress field, which opposes the volume change of the anatase to rutile phase transformation. 

Gennari and Pasquevich [65] have also demonstrated that the activation energy to nucleate rutile within anatase is larger when the sample is doped with a cation (Fe^3+^ originating from Fe_2_O_3_ in their case) but similar for crystallite growth. In this case, the phase transformation is diffusion-controlled and an increase in the cation concentration favors diffusion due to the creation of oxygen vacancies. As a consequence, the growth of rutile is enhanced, whilst the nucleation of anatase is inhibited. Note that the former work was carried out with powder samples which were initially composed of 95% of anatase, so only indirect correlations with this work can be made, since the initial structures and films are amorphous before the heat treatment. Moreover, lattice strain has also been reported to increase the anatase to rutile transformation temperature [59]. In detail, lattice strain can hinder crystallite growth and, thus, anatase crystallites will transform to rutile only after reaching a certain critical size. This size is, nonetheless, dependent on the fabrication route [59,61,62]. Kumar et al. has shown that the crystallite size of anatase in sol–gel-based films decreases as the alumina content is increased [62]. 

In summary, the introduction of aluminum cations [35] or alumina [61,62,67] is known to disturb the TiO_2_ unit cell by lattice distortion and also by the creation of oxygen vacancies. For compositions within the 16–32 wt.% range, the nucleation of crystallites is inhibited by the addition of Al^3+^ ions, whilst the growth of crystallites is inhibited by the lattice strain imposed by alumina clusters, and possibly also by the substrates [77]. Assuming a critical size, which needs to be reached for the crystallite to be stable and grow, we infer that for this middle range of compositions, this size is reached only at high temperatures in a condition where the formation of rutile is preferential due to the lower activation energy [59] and, thereby, anatase is not formed.

The compositional alumina–titania mixtures in which the crystallization occurs directly into the rutile phase, i.e., the formation of anatase is suppressed (Figure 4), are particularly interesting for application in photonic crystals used as reflectors, because the rutile phase is the crystal structure which presents the highest refractive index for titanium oxide. Moreover, the suppression of the anatase to rutile phase transition may be responsible for the improvement of thermal stability, as the phase transformation is known to induce coalescence and grain growth [22,63,66]. Such behavior is associated with the higher atomic mobility in the rutile phase [66]. A representative example is displayed in Figure 5e: after heat treatment at 900 °C, the sample presents a filter-like structure and the highly-ordered original shell structure is no longer observed in top view. Similar structures were obtained by Edelson and Glaeser [70] when sintering monodisperse titania submicrometric particles. Such filter-like structures were also reported for sintering of aluminum oxide ALD-based inverse opal photonic crystals but, in this case, heat-treated at a much higher temperature of 1400 °C for 4 h [19]. The formation of such a vermicular structure is associated with neck growth between ‘particle-like’ shells, but with inhibition of densification in some directions, causing the grain boundaries to be removed through the network, forming worm-like grains. This inhibition is caused, in normal powder metallurgy products and particle sintering, by the formation of pores which increase the distance between particle centers or inhibit particle contact [78]. The iPhCs here studied are highly porous structures, in which the macropores (former PS template) account for a maximum of 74% (theoretical FCC packing limit) of the structure. Moreover, the iPhC is composed of an ordered structure, by which the mass transport pathways are guided.

The destabilization of the pure titania iPhC starts already at 700 °C (Figure 5a), where cracks are observed throughout the sample. Meanwhile, the TiO_2_-Al_2_O_3_ and the Al_2_O_3_ iPhC structures (Figure 5b–d) are still stable and their structures resemble those after burn-out (Figure S4). Further annealing at 900 °C for 1 h promotes the destabilization of the titania iPhC structure due to sintering and grain growth (Figure 4e) [22]. Phase separation, which is in agreement with the phase diagram for both oxides [79], and nanosized cluster formation is observed for both TiO_2_-Al_2_O_3_ iPhCs (Figure 5f–g). Note that such an enhanced stability of the structure and ordering compared to pure titania iPhCs directly influences the optical properties of the alumina–titania mixed iPhCs after heat treatment, as discussed in the following section.

### 3.3. Optical Properties of Inverse Opal Photonic Crystals

**Figure 6:**
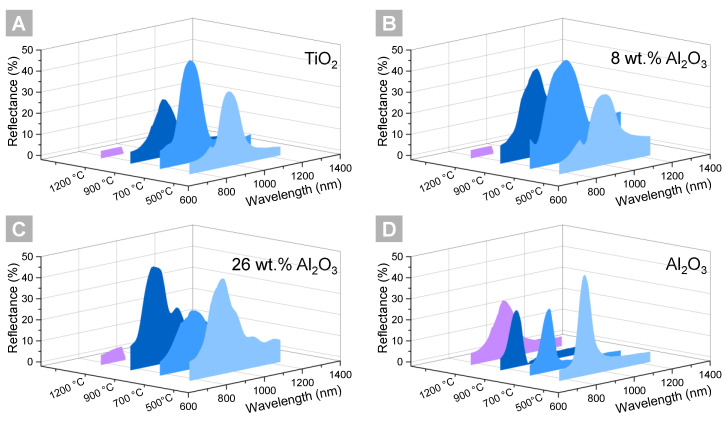
Specular reflectance of inverse photonic crystals according to the heat treatment temperature (500 to 1200 °C) for different materials: (**a**) TiO_2_ (**b**) TiO_2_ with 8 wt.% Al_2_O_3_ (**c**) TiO_2_ with 26% wt.% Al_2_O_3_ (**d**) Al_2_O_3_. The break observed in the data points at around 889 nm of wavelength is related to a mechanical change in the monochromator set up of the UV–Vis equipment.

The TiO_2_-Al_2_O_3_ iPhCs present a photonic bandgap even after heat treatment at 900 °C with no significant reduction in the reflectance (Figure 6b–c) compared to values after 700 °C heat treatment. Meanwhile, the TiO_2_ iPhCs (Figure 6a) present a severe reduction in the reflectance capability, associated with the structural destabilization discussed in the previous section. Despite the structure being clearly sintered, such iPhCs still present a bandgap, which is, at first, surprising. Nonetheless, we have already demonstrated in previous work that, despite the structural destabilization after heat treatment at 1000 °C in air, the internal structure still presents macropores in an ordered fashion, although grain nucleation and growth is clearly observed throughout the structure [22]. The Al_2_O_3_ iPhCs (Figure 6d) presented a photonic bandgap even after heat treatment at 1200 °C. Note that the reflectance capability of such structures is, however, lower at the conditions of heat treatment at 700 °C and 900 °C when compared to the mixed iPhCs. The position and intensity of a photonic bandgap in iPhCs is defined by several parameters [80], namely the macropore size (former PS template size), the shell material and surrounding material refractive indexes, the shell thickness, the interplanar spacing in relation to the incident light (d<111> for our samples), and the incident light angle (kept constant at 8° in our case). Moreover, it is also affected by the overall number of defects and the film thickness, defined during the self-assembly. In this work, all the iPhCs were assembled under the same run, inside the same humidity chamber, and the measurement area was kept constant at similar positions within the film. Thereby, the influence of self-assembly parameters is excluded.

A blue shift is observed in the photonic bandgap peak of the titania and alumina iPhCs after the first heat treatment at 700 °C, which accounts for 5% and 4%, respectively. The observed shift can be associated with two different factors: macropores’ shrinkage [20] and changes in the films’ refractive index (discussed in the section, 3.1. Refractive Index Evolution). The observed change in refractive index is minimal for alumina (1.66 to 1.64) but substantial for titania (2.16 to 2.28) due to crystallization (see Figure 2). Thus, the blue shift observed for alumina is related essentially to the macropores’ shrinkage, while the one in titania is a result of a larger blue shift caused by a pronounced macropore alteration counterbalanced with the red shift, which is expected due to the increase in the effective refractive index. A similar behavior is observed for the iPhC containing 8 wt.% of Al_2_O_3_. 

Interestingly, the two mixed phases, namely 8 wt.% and 26 wt.%, show different behavior when increasing the annealing temperature from 700 °C to 900 °C. Whereas the latter one shows a significant increase in reflection intensity, the reflection intensity of the iPhC with a lower alumina concentration remains almost constant. This observation agrees with the results discussed in previous sections: the refractive index for 8 wt.% alumina-containing titania thin films remains constant in this temperature range, whereas the one for the 26 wt.% mixture peaks at higher temperatures. Furthermore, the 26 wt.% composition crystallizes directly into rutile at temperatures around 700 °C. In contrast, the 8 wt.% iPhC crystallizes already at temperatures below 500 °C into the anatase phase and remains there even when higher heat treatment temperatures are applied.

It should be noted that the break in the data points observed at around 889 nm of wavelength is not related to the samples, i.e., it is not a second peak, but arises from the monochromator change during measurement.

To conclude this section, the analysis of the reflectance spectra proves that the introduction of alumina into the titania iPhC structure increases its stability up to 900 °C and, on account of this, the reflectance capability is increased compared to pure titania iPhCs.

## 4. Conclusions

This work demonstrates that the fabrication of multi-phase alumina–titania inverse opal photonic crystals in a wide range of compositions is possible by utilizing a super-cycle atomic layer deposition approach. Such tailor-made porous structures are potential candidates for applications such as oxygen sensors, photonic reflectors, and mechanical metamaterials, to name a few, depending on the alumina content. 

Specifically, the ALD films are amorphous after deposition and both the crystallization and phase transition temperatures of titania are affected by the alumina content. For the range of 16–32 wt.%, the formation of anatase is suppressed and the deposited thin films crystallize directly into the rutile phase. Moreover, the alumina–titania thin films and inverse opal photonic crystals showed enhanced thermal stability when compared to full titania without sacrificing the high refractive index property. 

We have explored the application of alumina-doped titania photonic crystals as high-temperature reflectors. The reflectance capability of the mixed iPhCs is kept constant even after exposure at 900 °C, and they outperform their pure titania and alumina counterparts. Finally, we demonstrate that tailoring the materials’ properties by supercyclic ALD processes on a sub-nm mixing scale is a versatile tool for solving a plethora of scientific challenges in material science and engineering.

## Supplementary Materials

The following are available online at https://www.mdpi.com/article/10.3390/nano11041053/s1, Figure S1. SEM top-view images of the films after annealing at 1200 °C showing destabilization and grain growth. Samples (a) T, (b) AT064, (c) AT032, (d) AT04, corresponding to estimated aluminum oxide content of 0, 8, 14 and 63%, respectively. Scale bars represent 1 µm; Figure S2. XRD ex situ measurements after annealing at 1200 °C showing that the samples are composed either of rutile phase only or of a mix between rutile (PDF pattern no. 01-072-7374) and anatase (PDF pattern no. 01-070-8501) phases. The respective phase and planes for each peak are identified, where ‘A’ stands for anatase and ‘R’ for rutile. Estimated aluminum oxide content is presented in Figure 1b. The peak at 2θ=21.4° for (a,f-k) corresponds to the main peak of cristobalite phase (PDF pattern no. 01-076-0934), which appears due to the crystallization of the silicon oxide layer of the silicon wafer. The reason that this peak is not visualized for every sample is associated with the film destabilization behavior, and thus different substrate exposure areas, shown in Figure S1; Figure S3. Full-version of XRD in situ measurements from RT up to 900 °C (0.25°C/s) and full-range measurements after 900 °C. Phases and the respective planes for each peak are shown, where ‘A’ stands for anatase (PDF pattern no. 01-070-8501) and ‘R’ for rutile (PDF pattern no. 01-072-7374). Estimated aluminum oxide content is presented in Figure 1b. Please note that the axes have slightly different ranges for (a-h) and (i-p). Reference sample with 100% aluminum oxide (i) presents peaks matching δ-alumina phase (PDF patterns no. 00-046-1131 and 00-046-1215). Selected compositions are also shown in Figure 3; Figure S4. Morphology of (a,b) TiO_2_ (c) TiO_2_-8 wt.% Al_2_O_3_ inverse opal photonic crystals imaged by SEM (inLens detector) in (a,c) top view and (b) cross-section view. Scale bars correspond to (a,c) 500 nm and (b) 2 µm.
