# Supplementary material for: Influence of Alumina Addition on the Optical Properties and the Thermal Stability of Titania Thin Films and Inverse Opals Produced by Atomic Layer Deposition
Source: Nanomaterials (Basel). 2021 Apr 20;11(4):1053. doi: 10.3390/nano11041053 (PMC8074236; doi:10.3390/nano11041053)
Supplement: Supplementary file 1 [file nanomaterials-11-01053-s001.zip › nanomaterials-1159049-supplementary.pdf]

nanomaterials

Supplementary Information

Influence of Alumina Addition on the Optical Properties and the Thermal Stability of Titania Thin Films and Inverse Opals Produced by Atomic Layer Deposition

Martin Waleczek [1], Jolien Dendooven [2], Pavel Dyachenko [3,4], Alexander Y. Petrov [4,5], Manfred Eich [4,5], Robert H. Blick [1], Christophe Detavernier [2], Kornelius Nielsch [6,7], Kaline P. Furlan [1,8,*] and Robert Zierold [1,*]

1 Institute of Nanostructure and Solid State Physics & Center for Hybrid Nanostructures, Universität Hamburg, Luruper Chausse 149, 22761 Hamburg, Germany; mwalecze@bynt.de (M.W.); rblick@physnet.uni-hamburg.de (R.H.B.)
2 Department of Solid State Sciences, COCOON Group, Ghent University, Krijgslaan 281/S1, B-9000 Ghent, Belgium; Jolien.Dendooven@UGent.be (J.D.); Christophe.Detavernier@UGent.be (C.D.)
3 Holoeye Photonics AG, Volmerstrasse 1, 12489 Berlin, Germany; p.n.dyachenko@gmail.com
4 Institute of Optical and Electronic Materials, Hamburg University of Technology, Eißendorfer Str. 38, 21073 Hamburg, Germany; a.petrov@tuhh.de (A.Y.P.); m.eich@tuhh.de (M.E.)
5 Institute of Hydrogen Technology, Helmholtz-Zentrum hereon, Max-Planck-Straße 1, D-21502 Geesthacht, Germany; a.petrov@tuhh.de (A.Y.P.); m.eich@tuhh.de (M.E.)
6 Institute of Materials Science, Technical University Dresden, Helmholtzstr. 10, 01069 Dresden, Germany; k.nielsch@ifw-dresden.de
7 IFW Dresden, Institute for Metallic Materials, Helmholtzstr. 20, 01069 Dresden, Germany
8 Institute of Advanced Ceramics, Hamburg University of Technology, Denickest. 15, 21073 Hamburg, Germany
* Correspondence: rzierold@physik.uni-hamburg.de (R.Z.); kaline.furlan@tuhh.de (K.P.F.)

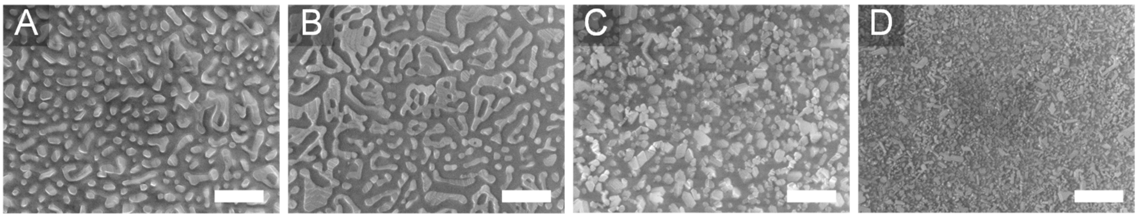

Figure S1. SEM top-view images of the films after annealing at 1200 °C showing destabilization and grain growth. Samples **(a)** T, **(b)** AT064, **(c)** AT032, **(d)** AT04, corresponding to estimated aluminum oxide content of 0, 8, 14 and 63%, respectively. Scale bars represent 1 µm.

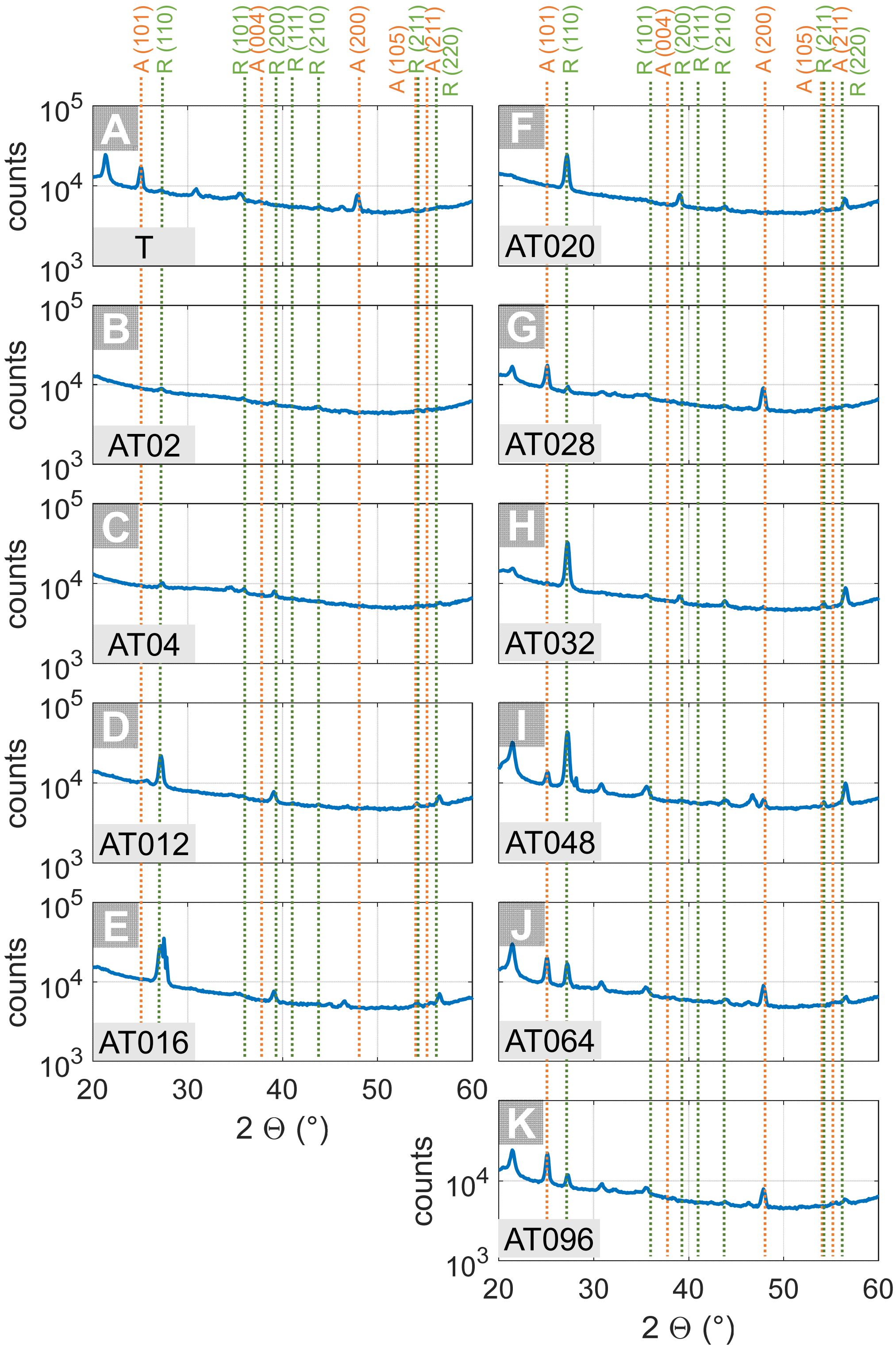

Figure S2. XRD *ex-situ* measurements after annealing at 1200 °C showing that the samples are comprised either by rutile phase only or by a mix between rutile (PDF pattern no. 01-072-7374) and anatase (PDF pattern no. 01-070-8501) phases. The respective phase and planes for each peak are identified, where 'A' stands for anatase and 'R' for rutile. Estimated aluminum oxide content is presented at Figure 1b. The peak at 2θ = 21.4° for (a,f–k) corresponds to the main peak of cristobalite phase (PDF pattern no. 01-076-0934), which appears due to the crystallization of the silicon oxide layer of the silicon wafer. The reason why this peak is not visualized for every sample is associated to the film destabilization behavior, and thus different substrate exposure areas, shown in Figure S1.

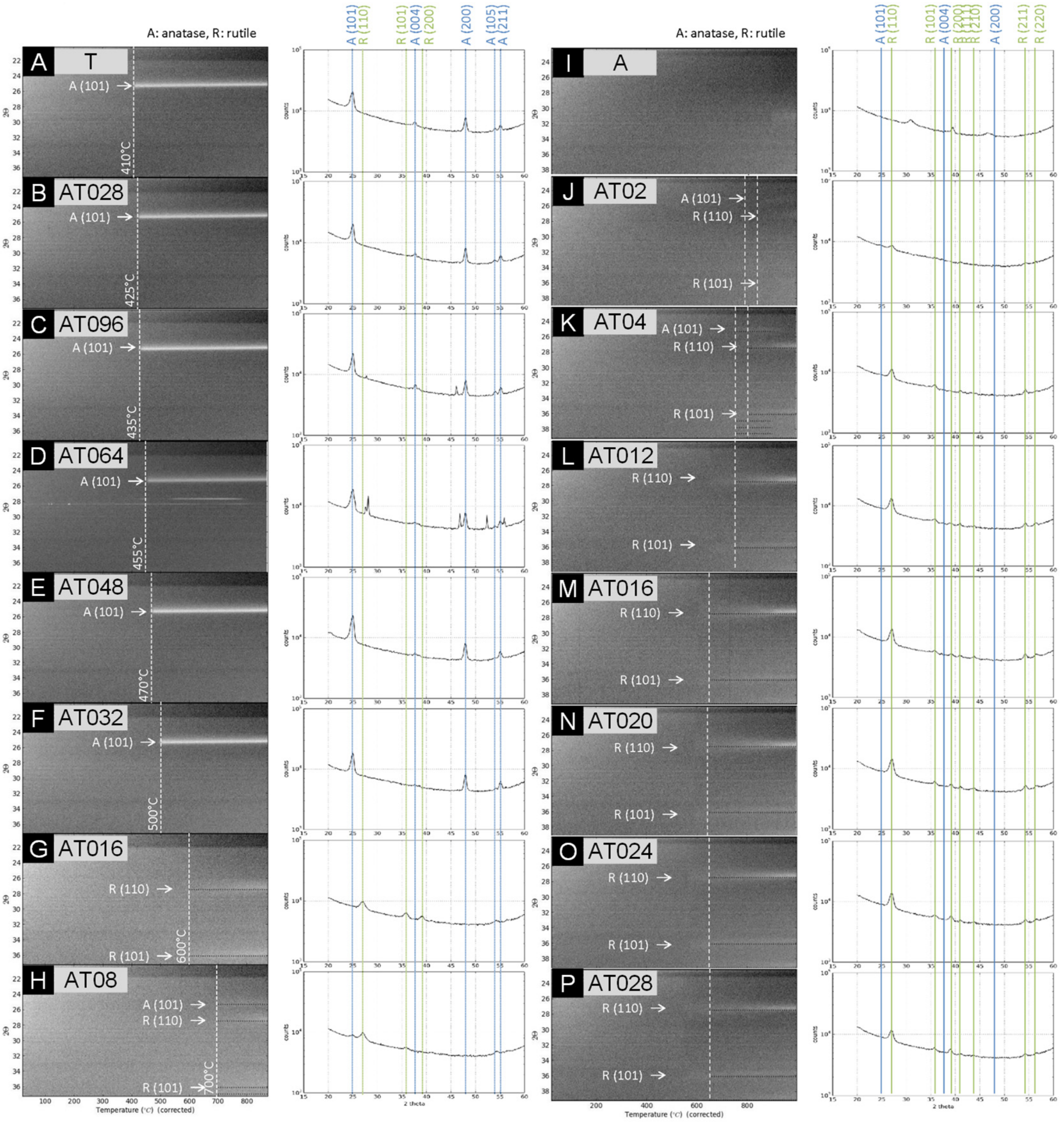

Figure S3. Full-version of XRD in-situ measurements from RT up to 900 °C (0.25 °C/s) and full-range measurements after 900 °C. Phases and the respective planes for each peak are shown, where 'A' stands for anatase (PDF pattern no. 01-070-8501) and 'R' for rutile (PDF pattern no. 01-072-7374). Estimated aluminum oxide content is presented at Figure 1b. Please notice that the axes have slightly different range for **(a–h)** and **(i–p).** Reference sample with 100% aluminum oxide (i) presents peaks matching δ-alumina phase (PDF patterns no. 00-046-1131 and 00-046-1215). Selected compositions are also shown in Figure 3.

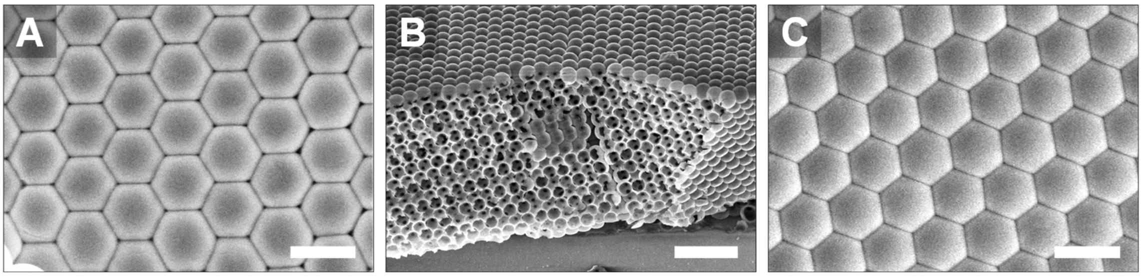

Figure S4. Morphology of **(a,b)** TiO_2 **(c)** TiO_2-8 wt.% Al_2O_3 inverse opal photonic crystals imaged by SEM (inLens detector) in (a,c) top view and (b) cross section view. Scale bars correspond to (a,c) 500 nm and (b) 2 µm.
